# miR-125b-5p upregulation by TRIM28 induces cisplatin resistance in non-small cell lung cancer through CREB1 inhibition

**DOI:** 10.1186/s12890-022-02272-9

**Published:** 2022-12-07

**Authors:** Qiuyu Tan, Jinzhu Ma, Hao Zhang, Xu Wu, Qiang Li, Xiaoxuan Zuo, Yuxin Jiang, Haijun Liu, Liang Yan

**Affiliations:** 1 The First Affiliated Hospital of Wannan Medical College (Yijishan Hospital of Wannan Medical College), Wuhu, 241002 Anhui China; 2 Provincial Key Laboratory of Biological Macro-Molecules Research, Wannan Medical College, Wuhu, 241002 Anhui China; 3 Department of Pathogen Biology and Immunology, Jiaxing University College of Medicine, Jiaxing, 314000 Zhejiang China; 4 Department of Orthopedics, Changhai Hospital, Navy Medical University, Shanghai, 200433 China

**Keywords:** miR-125b-5p, CREB1, DDP resistance, TRIM28, Non-small cell lung cancer

## Abstract

**Objective:**

miR-125b-5p plays an important role in the development of cancer and drug resistance. However, in cisplatin resistance of non-small cell lung cancer (NSCLC), the function and potential mechanism of miR-125b-5p is still unclear. The aim of this study was to investigate the role and molecular mechanism of miR-125b-5p in cisplatin resistance of NSCLC.

**Methods:**

A GEO dataset (GSE168707) was analyzed to find high miR-125b-5p levels were associated with DDP resistance. miR-125b-5p expression levels were detected in A549 and A549/DDP cells via real-time quantitative RT-PCR (qRT-PCR). Luciferase reporter assays, western blots and mouse model xenografted were performed to identify CREB1 as a direct target gene of miR-125b-5p. Cell proliferation and apoptosis were also performed to identify whether miR-125b-5p upregulation by TRIM28 induces DDP resistance in NSCLC through CREB1 inhibition.

**Results:**

In A549/DDP cells, miR-125b-5p expression was upregulated compared to A549 cells. Then miR-125b-5p was found to increase DDP resistance in NSCLC in vivo and in vitro by increasing cell proliferation and suppressing cell apoptosis. Bioinformatic analyses were used to search for gene which miR-125b-5p can target. We identified miR-125b-5p can regulate CREB1 via luciferase reporter assays, qRT-PCR and western blots. Cell proliferation and apoptosis were also performed to confirm miR-125b-5p could impact on CREB1 and induce the DDP resistance in NSCLC. Additionally, we used bioinformatic analyses to find tripartite motif-containing 28 (TRIM28) as a transcriptional enhance factor of miR-125b-5p. The expression of TRIM28 was upregulated in A549/DDP cells compared with that in A549 cells by qRT-PCR. Finally, we found TRIM28 could mediate DDP resistance through miR-125b-5p/CREB1 axis via cell proliferation, western blot and apoptosis assay.

**Conclusions:**

Overall, our findings demonstrated novel functions and mechanisms underlying DDP resistance in NSCLC through the TRIM28/miR-125b-5p/CREB1 axis. These may serve as novel therapeutic targets to improve the treatment efficacy using DDP for NSCLC in the future.

**Supplementary Information:**

The online version contains supplementary material available at 10.1186/s12890-022-02272-9.

## Introduction

Lung cancer (LC) is a malignant tumor with the high incidence rate and mortality worldwide, whereby 80% of all LC cases are those of non-small cell LC (NSCLC) [1]. Surgery is performed for patients with early NSCLC but chemotherapy is the one of treatment options currently available for metastatic and advanced cases [2]. Cisplatin (DDP), which can induce apoptosis through DNA damage, is an effective chemotherapeutic drug commonly used in the clinical treatment of NSCLC [3]. However, following long-term use, these patients often develop resistance to DDP, thus affecting the chemotherapeutic efficacy [4, 5]. Understanding the mechanism underlying DDP resistance may provide clues for new treatment efforts aimed at improving the efficacy of chemotherapy for advanced and metastatic NSCLC.

MicroRNAs (miRNAs) are non-coding RNA (ncRNAs) that are 18–24 nucleotides long. Owing to sequence complementarity, these RNAs bind to the 3′ untranslated region (3′UTR) of the target mRNA, thus inhibiting the translation of the target gene [6, 7]. Accumulating data show that miRNAs exert critical regulatory effects on chemoresistance in LC [8]. For example, miR-30a-5p combined with gemcitabine overcomes acquired resistance in NSCLC cells [9]. miR-193a derived from exosomes of bone marrow mesenchymal stem cells induces DDP resistance in NSCLC cells [10]. Previous evidence demonstrates the function of miR-125b-5p in suppressing several tumor types, including pancreatic and breast cancers [11, 12]. Additionally, miR-125b-5p enhances the sensitivity of gall bladder cancer cells to DDP resistance by suppressing the expression of Bcl-2 [13]. Nevertheless, in NSCLC, the role of miR-125b-5p in DDP resistance remains unknown and warrants investigation.

cAMP response element-binding protein 1 (CREB1) is a DNA binding protein of the leucine zipper family. It is a regulatory factor in the nucleus of eukaryotic cells [14]. Previous studies show that CREB1 plays an important role in several types of cancer [15]. CREB1 affects cell proliferation, apoptosis, and migration of NSCLC cells [16]. Recently, Kim et al. showed that CREB1 inactivation induced DDP resistance in NSCLC by attenuating the activation of ERK [17]. The mechanism of resistance of NSCLC cells to platinum compounds remains unclear. Moreover, the underlying mechanism of action remains poorly understood, and further research is needed.

In this study, we analyzed the datasets from the gene expression omnibus (GEO) database and found significantly higher miR-125b-5p levels in specimens that were DDP-resistant than DDP-sensitive specimens. miR-125b-5p levels were remarkably higher in A549/DDP cells as compared to the A549 cells. Through bioinformatic analysis, miR-125b-5p was found to directly target CREB1. Subsequently, miR-125b-5p was found to induce DDP resistance by attenuating CREB1 expression in NSCLC cells. Then miR-125b-5p could induce the resistance of NSCLC xenografts to DDP by targeting CREB1 in vivo. Finally, TRIM28 induced DDP resistance in NSCLC by upregulating miR-125b-5p levels. Overall, in NSCLC, the TRIM28/miR-125b-5p/CREB1 axis underlies a crucial mechanism of DDP resistance.

## Materials and methods

### Bioinformatics analysis of differentially expressed (DE) miRNAs

Microarray data of microRNA expression profiles associated with cisplatin-sensitive/resistant were retrieved from the gene expression omnibus (GEO) database (https://www.ncbi.nlm.nih.gov/geo/). Subsequently, we downloaded the compliant microRNA expression dataset GSE168707 (https://www.ncbi.nlm.nih.gov/geo/query/acc.cgi?acc=GSE168707). The dataset was based on the GPL29837 platform and contained 6 lung tissue samples, including 3 each from cisplatin-sensitive and cisplatin-resistant lung cancer patients. Screening of DE-microRNAs was performed using GEO2R (https://www.ncbi.nlm.nih.gov/geo/geo2r/) with the criteria of adj.*p*.val < 0.01 and |log2 fold change (FC)| > 1. Finally, volcano plots were used to visualize DE-microRNAs.

### Cell culture

Human LC cell lines A549 was purchased from Cell Bank of Chinese Academy of Sciences (Shanghai, China), and human lung adenocarcinoma drug-resistant cell line (A549/DDP) was purchased from Fuheng Biotechnology Co., Ltd (Shanghai, China). The cells were grown in DMEM (Hyclone) supplemented with 10% fetal bovine serum at 37 °C with 5% CO_2_.

### Cell transfection

The inhibitors/mimics of miR-125b-5p together with corresponding control mimics/inhibitors were procured from Ribobio (Guangzhou, China). The siRNA of CREB1 and control siRNAs were procured from Genepharma (Shanghai, China). The overexpressed CREB1 and control plasmids were procured from Genecopoeia (Guangzhou, China). These miRNA mimics, inhibitors, siRNAs, and CREB1 overexpressed plasmid were processed using lipo3000 (Invitrogen, CA) following the manufacturer’s specification.

### Cell viability

The Cell Counting Kit 8 was used to detect cell viability (Beyotime, China). The transfected cells were inoculated on the 96-well plate. Following the attachment of the cells to the walls, these were incubated with different indicated concentrations of DDP (0 and 6.25 μg/mL). Following this, the cells were incubated supplemented with the CCK8 working solution for 2 h, and the OD was obtained at 450 nm using a spectrophotometer.

### Cell apoptosis assays

Annexin V-propyl iodide (PI) (Keygen, China) staining was performed to measure the rate of apoptosis following the kit specifications. The cells were incubated with different concentrations of DDP (0 and 6.25 μg/mL) for another 24 h following transfection. The upper culture fluid and cells were acquired, and the protocol was followed. The cells were analyzed by Beckman Coulter flow cytometry.

### Protein extraction and western blot

RIPA (Beyotime, China) was used for extracting proteins from cells. The expression levels of CREB1 and TRIM28 were evaluated by western blotting. For protein isolation, 10% SDS-PAGE (Bio-Rad) was used, then the protein was transfered to Polyvinylidene fluoride (PVDF) membrane. To prevent non-specific binding, the membranes were placed in Tris-buffered saline-Tween-20 containing 5% skimmed milk at room temperature for 1 h. According to the molecular weight of protein in the instruction manual and the pre-experiment results, we cut the membrane. After that, the primary antibodies against CREB1 CREB1 (Cat No. A1189, 1:1000, ABclonal, Wuhan, China), TRIM28 (Cat No. 66630-1-Ig, 1:2000, Proteintech, Wuhan, China), and GAPDH (Cat No. 5174S, 1:2000, Cell Signaling Technology, USA) were added into the membranes for overnight at 4 ℃. The membranes were cleaned and incubated with goat anti-rabbit secondary antibody (Cat No. sc-2005, 1:5000, Santa, TX, USA) at room temperature for 1 h. Finally, we use the chemiluminescence working solution (Thermo, CA, USA) to visualize and take images. GAPDH was the internal reference used to compare the relative levels of CREB1 expression. The band intensities were analyzed using Image J.

### Configuration of DDP

DDP (Hansoh Pharma, Lianyungang, China) was mixed into DMEM supplemented with 2% fetal bovine serum to achieve final concentrations of 6.25 μg/mL. DDP was first blended into PBS and injected intraperitoneally into mice for DDP treatment.

### Mouse model xenografted with A549 cells

Ten mice (female, BALB/c-nude) were the experimental animals in this study and were procured from GemPharmatech (Nanjing, China). They were reared in Wannan Medical College’s SPF laboratory animal room. The Ethics Committee of Wannan Medical College approved (NO. LLSC-2020-030) the experimental design. A follow-up experiment was performed a week after the mice got acclimatized. A549 cells with either overexpressing miR-125b-5p lentiviruses or empty lentiviruses (1 × 10^7^ cells per mouse; 5 mice per group) were subcutaneously injected. The empty lentiviruses were as the control group. Three weeks after subcutaneous tumor implantation, the nude mice were treated with DDP (5 mg/Kg administered for two weeks). Subsequently, the mice were euthanized with a CO_2_ overdose and the tumor tissue was removed. The samples were fixed with 4% paraformaldehyde; subsequently, hematoxylin and eosin (H&E) and immunohistochemical staining assays were performed. Western blotting and qRT-PCR were also performed using the tumor tissue samples.

### qRT-PCR

Following the manufacturer′s protocol, total RNA from the cells was extracted and reverse-transcribed into cDNA. Levels of miR-125b-5p (ID 000449, Thermo, USA) were detected by the Taqman probe method and those of CREB1, TRIM28, GATA2, CHD2.

RAB21 and POLR2A mRNA by SYBR Green Master Mix. qRT-PCR was conducted on the Applied Biosystems 7500 equipment using Invitrogen SYBR green dye or TaqMan. The sequences of primers used in this study are listed in Table 1. The 2^−ΔΔCt^ method was utilized for normalizing the levels of CREB1, TRIM28, GATA2, CHD2.

**Table 1:** Sequences of primers

Primer	Forward	Reverse
CREB1	5′-CAGCCTCCGGACTCTAGC-3′	5′-TAATACGACTCACTATAGGG-3′
GATA2	5′-CTGCCGCCACATCCATCCT-3′	5′-TGCAGACGGCAACGGC-3′
CHD2	5′-TTGCATTGACAGCTTCCACAG-3′	5′-CCTAGGCCCATTTCATCAGC-3′
RAD21	5′-CCCGTTGAACCAATGCCAAC-3′	5′-TCAATTTCTTGGTGGGCGGT-3′
POLR2A	5′-TGCTGGTTTTGGTGACGACT-3′	5′-TCTGTCTGTGGCAAGTGCAT-3′
TRIM28	5′-CAGCTACTGTGTGGAGTGCT-3′	5′-CGTTCACCATCCCGAGACTT-3′

RAB21 and POLR2A expression to GAPDH. And the 2^−ΔΔCt^ method was utilized for normalizing the levels of miR-125b-5p expression to U6 (ID 001973, Thermo, USA).

### Luciferase reporter assays

A luciferase reporter assay was performed to check if miR-125b-5p could bind to CREB1. Sequences binding to miR-125b-5p on CREB1 3'UTR were deleted to obtain the mutant. The CREB1 3′UTR wild-type and mutant sequences were inserted into the p-MIR-reporter (Ambion, Austin). The wild-types and mutant sequences were confirmed by sequencing. Subsequently, in 24-well plates, the 293T cells were seeded. At 70% confluency, the same amount of β-galactosidase (Ambion) and luciferase firefly reporter plasmids were transfected, with an equal amount of miR-125b-5p mimic/control mimic. Following a previously reported protocol, we performed luciferase assays [18].

### Statistical analysis

The results of the statistical analysis were expressed as mean +/− SD. These were obtained from at least three independent experimental repeats. The Turkish trial was used for postmortem analysis, with several comparative tests. *p* < 0.05 denoted statistical significance.

## Results

### miR-125b-5p expression is upregulated in A549/DDP cells

**Fig. 1:**
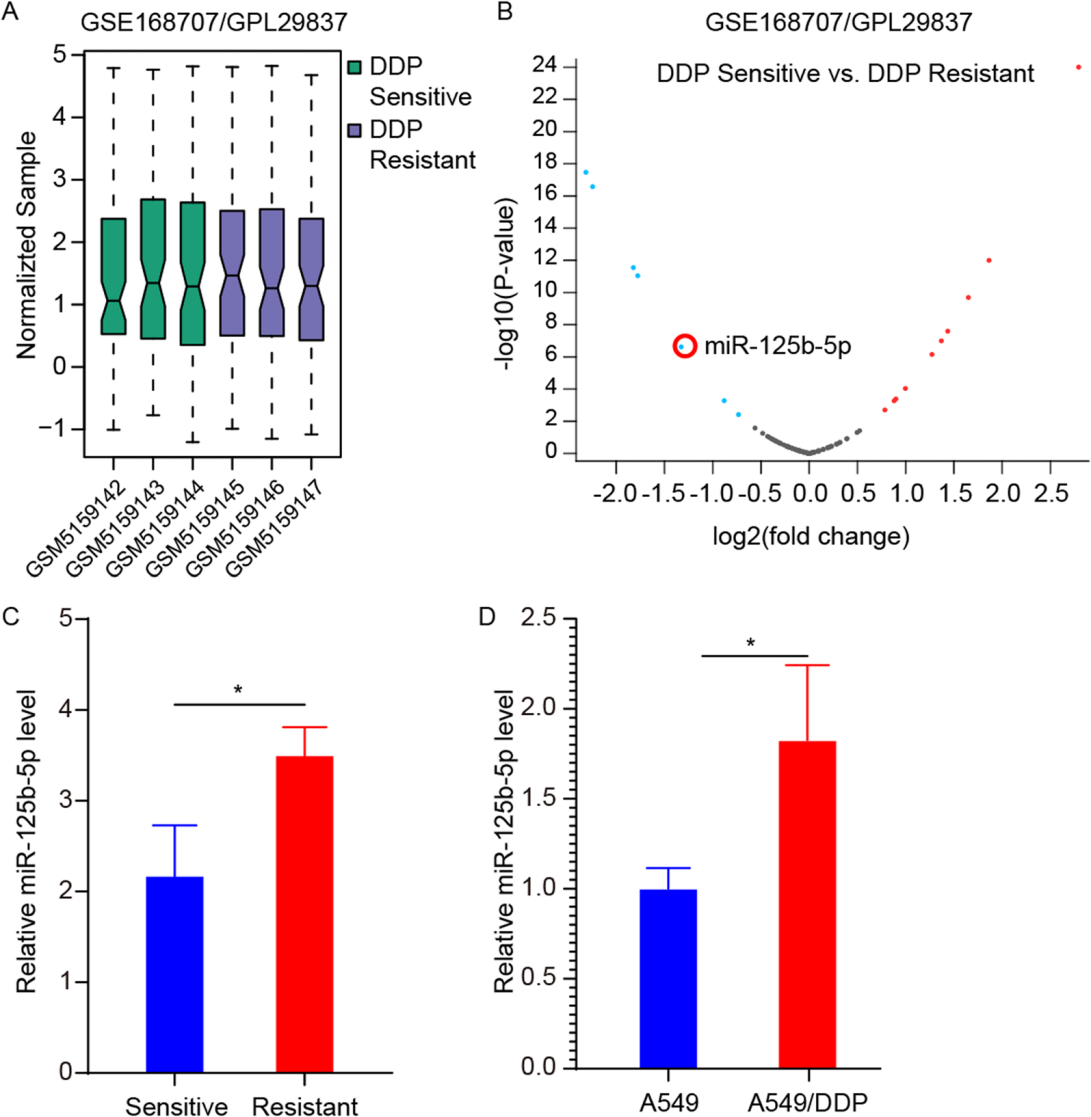
miR-125b-5p expression is upregulated in DDP-resistant LC patients and A549/DDP cells. **A** Data normalization for DDP-sensitive and DDP-resistant lung cancer samples. **B** Volcano plot of differentially expressed miRNAs, red circles indicate the target molecule miR-125b-5p in this study. **C** The relative miR-125b-5p expression was examined in DDP-sensitive and DDP-resistant patients in the GEO database. **D** miR-125b-5p expression was determined in A549 and A549/DDP cells by qRT-PCR

A GEO dataset (GSE168707) was analyzed to assess miR-125b-5p’s involvement in DDP resistance in NSCLC. Levels of miR-125b-5p were remarkably higher in DDP-resistant patients relative to those who were DDP-sensitive (Fig. 1A–C). miR-125b-5p levels were higher in A549/DDP cells relative to A549 cells (Fig. 1D). The levels of miR-125b-5p were remarkably high in A549/DDP cells. In NSCLC cells, high miR-125b-5p levels were associated with DDP resistance, demonstrating that miR-125b-5p could be a potential treatment target to overcome DDP resistance.

### In NSCLC, miR-125b-5p reduces the sensitivity to DDP, leading to DDP resistance

**Fig. 2:**
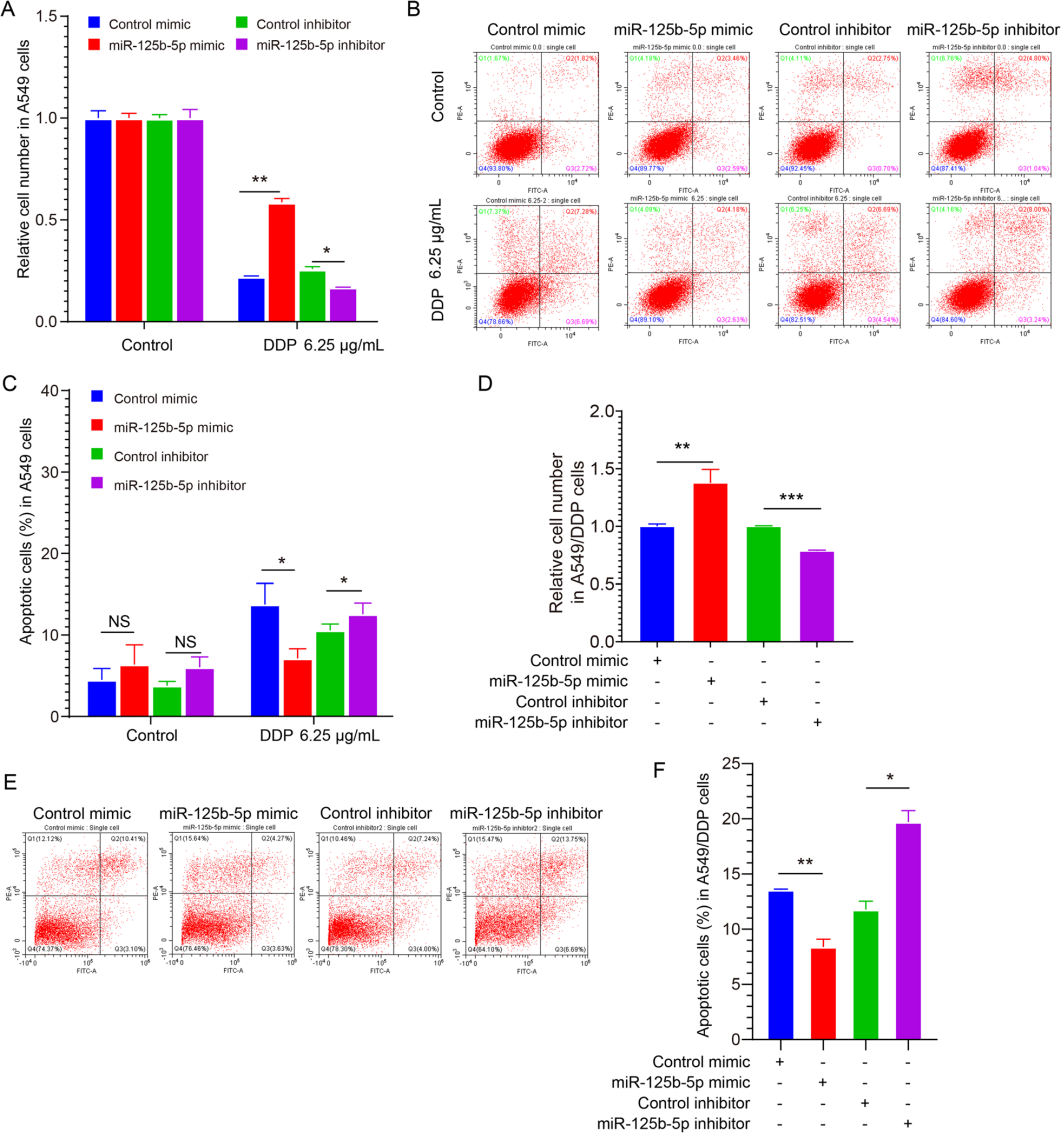
miR-125b-5p reduces the sensitivity of NSCLC cells to DDP, thus leading to DDP resistance. **A** CCK8 assay was performed to detect DDP sensitivity in miR-125b-5p mimic/inhibitor-treated A549 cells upon stimulation with the indicated concentrations of DDP (0 μg/mL, 6.25 μg/mL). **B**, **C** Apoptosis analysis in A549 cells following transfection using miR-125b-5p mimic/inhibitor upon treatment with the indicated concentrations of DDP (0 μg/mL, 6.25 μg/mL). **D** CCK8 assay was performed to detect DDP resistance in miR-125b-5p mimic/inhibitor-treated A549/DDP cells upon stimulation with the indicated concentrations of DDP (0 μg/mL, 6.25 μg/mL). **E**, **F** Apoptosis analysis in A549/DDP cells following transfection with miR-125b-5p mimic/inhibitor upon treatment with the indicated concentrations of DDP. *p < 0.05, **p < 0.01, ***p < 0.05

We knocked down or overexpressed miR-125b-5p to investigate whether changes in its levels in A549 cells affected cell sensitivity to DDP. Subsequently, the viability of A549 cells upon treatment was assessed by the CCK-8 assay for various doses of DDP (0, 6.25 μg/mL). The findings suggested that in the miR-125b-5p mimic group, the cell viability of A549 following stimulation by DDP was substantially elevated in contrast with the control mimic group (Fig. 2A). On the contrary, in the miR-125b-5p inhibitor group, the cell viability was considerably attenuated (Fig. 2A) as opposed to the control inhibitor group. Additionally, A549 cell apoptosis was detected upon stimulation with indicated DDP concentrations. The apoptosis rate in A549 cells in the miR-125b-5p mimic group reduced significantly relative to the control, whereas a significantly attenuated apoptosis was observed upon miR-125b-5p inhibitor treatment in A549 cells (Fig. 2B, C). Thus, miR-125b-5p reduced the sensitivity of A549 cells to DDP.

Next, miR-125b-5p was knocked down or overexpressed in A549/DDP cells to confirm whether, in NSCLC, a change in DDP sensitivity induced by miR-125b-5p led to DDP resistance. A CCK-8 assay was conducted to test the viability of A549 cells. These findings indicated that in the miR-125b-5p mimic group, A549 cellular viability was substantially elevated in contrast with the control mimic group (Fig. 2D). On the contrary, the cell viability upon treatment with the miR-125b-5p inhibitor was attenuated substantially (Fig. 2D) in comparison with the control inhibitor group. The results suggested that the rate of apoptosis of A549 cells in the miR-125b-5p mimic group reduced remarkably in comparison with the control (Fig. 2E, F), while that of A549 cells in the miR-125b-5p inhibitor group was enhanced significantly (Fig. 2E, F). Thus, miR-125b-5p attenuated A549 cells’ sensitivity to DDP, which induced DDP resistance in NSCLC cells.

### Prediction and demonstration of CREB1 as the direct target of miR-125b-5p

**Fig. 3:**
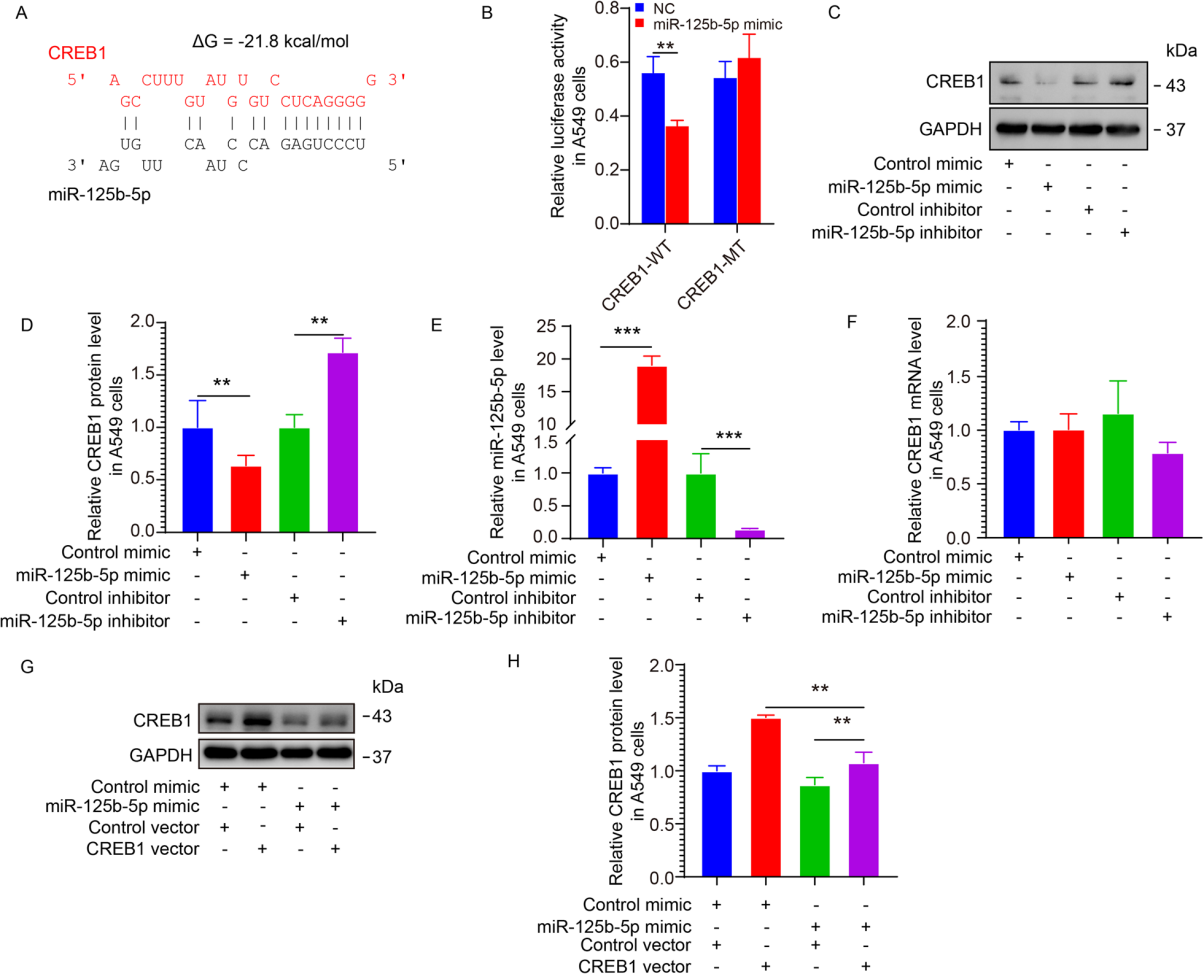
Prediction and demonstration of CREB1 as the direct target of miR-125b-5p. **A** Bioinformatic analysis for miR-125b-5p binding to CREB1 3′UTR. **B** Luciferase reporter plasmids carrying CREB1 3'UTR and wild-type/mutant miR-125b-5p binding regions were transfected with the same amount of control mimic or miR-125b-5p mimic constructs. **C**–**F** After transfecting A549 cells with miR-125b-5p mimic/inhibitor, CREB1 protein expression was quantified by western blotting (**C**, **D**); miR-125b-5p and CREB1 mRNA expression levels were analyzed by qRT-PCR (**E**, **F**). **G** and **H** The protein expression of CREB1 was quantified by western blotting for the following conditions: a. control mimic + control vector; b. control mimic + CREB1 vector; c. miR-125b-5p mimic + control vector; d. miR-125b-5p mimic + CREB1 vector. **p < 0.01, ***p < 0.001

To identify the possible binding target genes of miR-125b-5p, three computer applications (Targetscan V8.0 https://www.targetscan.org/vert_80/, starBase V2.0 https://starbase.sysu.edu.cn/starbase2/, and Pictar https://pictar.mdc-berlin.de/) were used [19]. Among these candidate genes, CREB1 was consistently predicted by all three applications. Figure 3A shows the possible arrangement of miR-125b-5p binding to CREB1. The binding energy of miR-125b-5p to CREB1 3'UTR was − 21.8 kcal/mol, indicating a feasible interaction between them. As illustrated in Fig. 3B, the dual-luciferase reporter assay confirmed a direct binding between miR-125b-5p and CREB1 3′UTR.

To demonstrate that miR-125b-5p mediated the expression of CREB1, we regulated the expression of miR-125b-5p in A549 cells. Upon miR-125b-5p upregulation (Fig. 3C), the expression of CREB1 protein was downregulated (Fig. 3D, E). Conversely, upon miR-125b-5p inhibition (Fig. 3C), CREB1 protein was upregulated (Fig. 3D, E). However, miR-125b-5p overexpression and suppression did not impact the mRNA expression of CREB1 (Fig. 3F). These results showed that CREB1 was indeed the directly targeted gene by miR-125b-5p and its expression was affected by miR-125b-5p post-transcriptionally.

To verify that miR-125b-5p modulated CREB1 expression, a rescue experiment was performed. Next, the protein levels of CREB1 were assessed by western blotting. As shown in Fig. 3G, H, overexpression of miR-125b-5p restored the effects of CREB1 upregulation. Thus, miR-125b-5p negatively elevated CREB1 levels post-transcriptionally.

### miR-125b-5p induces resistance to DDP by downregulating CREB1 expression in NSCLC cells

A recent study demonstrates that a decrease in the phosphorylation level of CREB1 causes DDP resistance in LC cells [17]. To investigate whether CREB1 levels were reduced in DDP-resistant NSCLC cells, we first determined CREB1 expression between A549 and A549/DDP via western blot analysis. CREB1 protein levels were low in A549/DDP as compared to A549 (Additional file 1: Fig. 1A, B). Further, we knocked down or overexpressed CREB1 in A549/DDP cells. Western blotting indicated that knocking down or overexpressing CREB1 in A549/DDP cells caused suppression (Additional file 1: Fig. 1C, D) or elevation (Additional file 1: Fig. 1E, F) in CREB1 protein levels, respectively. The findings of the CCK8 assay showed that A549/DDP cell viability in the Si-CREB1 group was substantially elevated relative to the Si-Control group (Additional file 1: Fig. 1G); On contrary, cell viability in the CREB1 overexpression group was considerably attenuated (Additional file 1: Fig. 1G) as opposed to the Control group. Additionally, we detected apoptosis in A549/DDP. The apoptosis rate in A549/DDP cells in the CREB1 knockout group reduced substantially relative to that in the control group (Additional file 1: Fig. 1H, I), while it was enhanced in the CREB1 vector group (Additional file 1: Fig. 1H, I). Therefore, in NSCLC cells, CREB1 could inhibit the resistance of DDP.

**Fig. 4:**
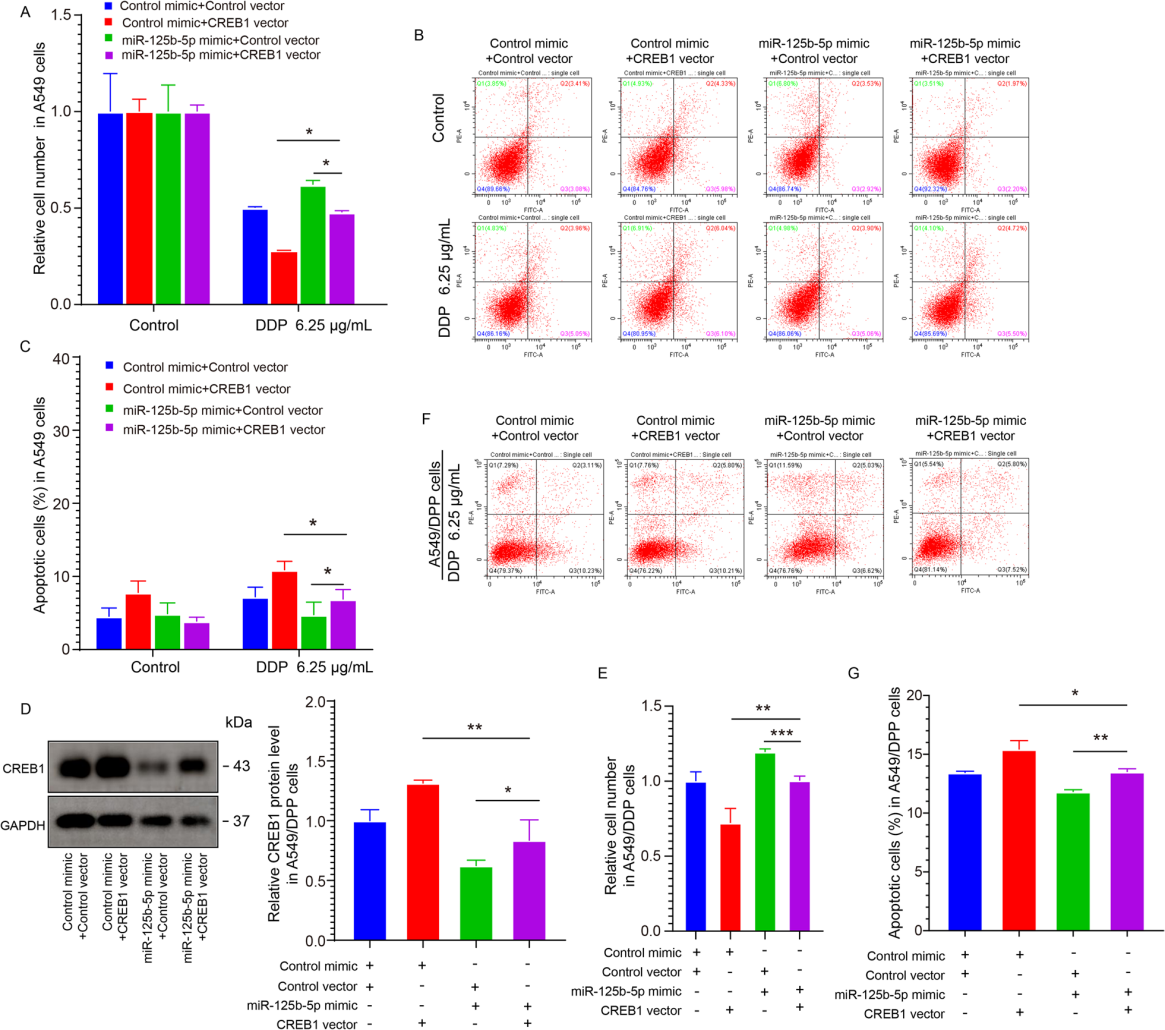
miR-125b-5p induces resistance to DDP by down-regulating CREB1 expression in NSCLC cells. **A** CCK8 assay was performed to detect DDP sensitivity in A549 cells upon stimulation with indicated concentrations of DDP (0 μg/mL, 6.25 μg/mL) under the following conditions: a. control mimic + control vector; b. control mimic + CREB1 vector; c. miR-125b-5p mimic + control vector; d. miR-125b-5p mimic + CREB1 vector. **B**, **C** Apoptosis analysis in A549 cells following transfection conditions described in A at indicated concentrations of DDP. **D** CREB1 protein expression was quantified by western blotting for A549/DDP cells following transfection described in **A**. **E** CCK8 assay was performed to detect DDP resistance in A549/DDP cells following transfection described in A. **F**, **G** Apoptosis analysis in A549/DDP cells following transfection described in **A**. *p < 0.05, **p < 0.01, ***p < 0.001

To prove that miR-125b-5p negatively regulated CREB1 expression, four different groups were transfected in A549/DDP and A549 cells as follows: a. Control vector + Control mimic; b. CREB1 vector + Control mimic; c. Control vector + miR-125b-5p mimic, and d. CREB1 vector + miR-125b-5p mimic. As shown in Fig. 4A–G, the cellular viability in the miR-125b-5p mimic + CREB1 vector group was between those in the CREB1 vector and miR-125b-5p mimic groups (Fig. 4A) following stimulation by DDP. miR-125b-5p overexpression could reverse the effects of CREB1, thereby confirming that miR-125b-5p reduced DDP sensitivity in A549 cells via the mechanism of targeting CREB1. Additionally, apoptosis assays also confirmed the above result (Fig. 4B, C). Next, the protein levels of CREB1 were assessed by western blotting. As shown in Fig. 4D, overexpression of miR-125b-5p restored the effects of CREB1 upregulation in A549/DDP cells. Then the viability of A549/DDP cells transfected with the miR-125b-5p mimic + CREB1 vector was between to that in the CREB1 vector and miR-125b-5p mimic group (Fig. 4E). Additionally, apoptosis assays showed that miR-125b-5p indeed induced A549/DDP cells’ resistance to DDP by targeting CREB1 (Fig. 4F, G). miR-125b-5p overexpression could thus reverse CREB1’s effects, thereby proving that miR-125b-5p induced cells’ resistance to DDP by targeting CREB1. The above results demonstrated in NSCLC cells that miR-125b-5p induced resistance to DDP by downregulating CREB1.

### miR-125b-5p induces in vivo DDP resistance by targeting CREB1

**Fig. 5:**
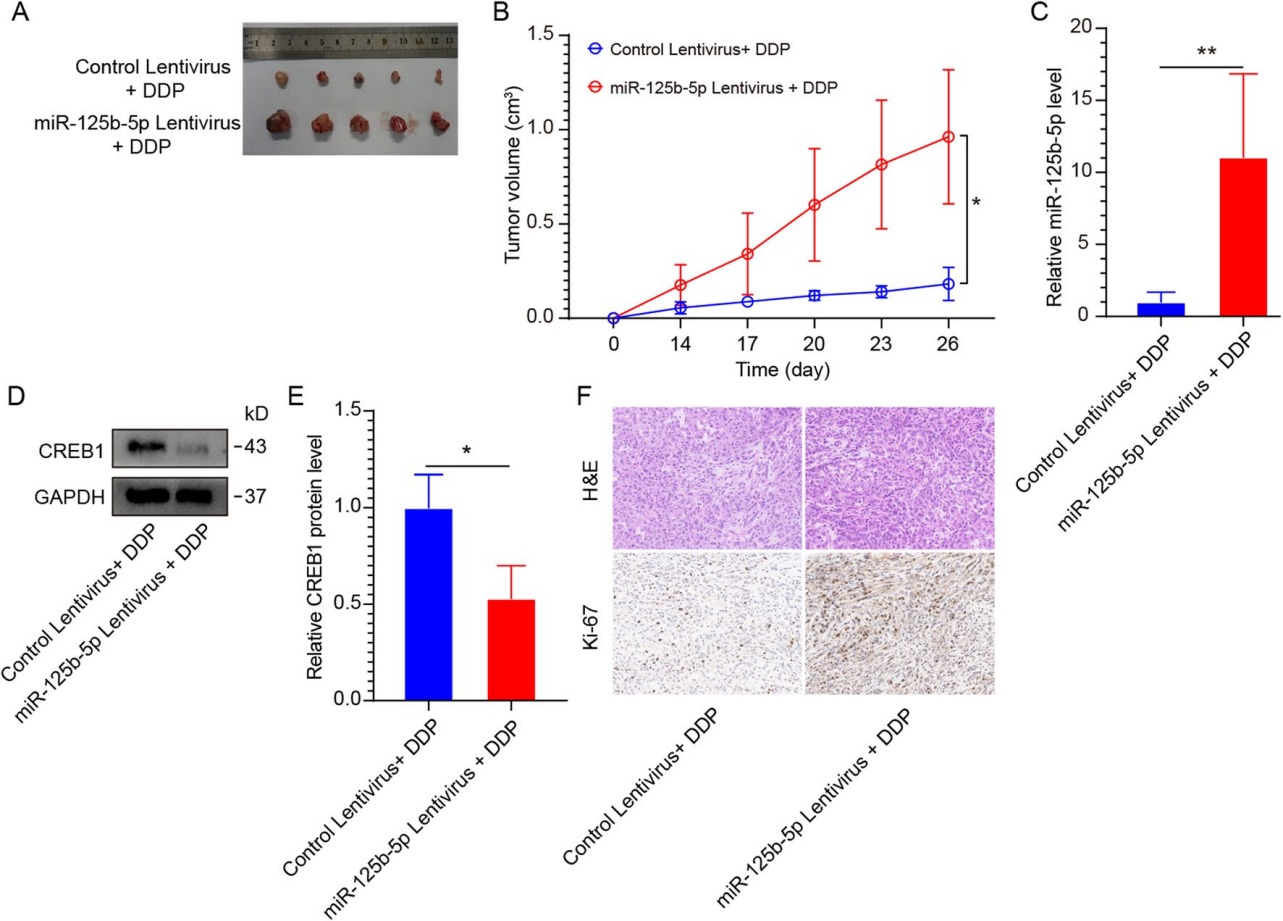
miR-125b-5p lessens the sensitivity to DDP in vivo. **A** Representative images of xenografts generated by transfecting A549 cells using miR-125b-5p or empty lentiviruses. **B** Growth curve for xenograft produced by A549 cells with miR-125b-5p or empty lentiviruses in vivo. **C** Analysis of miR-125b-5p expression in xenografts by qRT-PCR. **D** CREB1 protein levels in xenografts by western blotting. **E**, **F** H&E staining and immunohistochemistry to detect pathological changes in xenografts. *p < 0.05; **p < 0.01

We subcutaneously injected A549 cells carrying a lentiviral vector overexpressing miR-125b-5p or a corresponding control vector (1 × 10^5^ cells/200 μl per mouse) into the armpit of nude mice to construct a nude mouse xenograft model for verifying the in vivo impact of miR-125b-5p on the sensitivity to DDP. Three weeks later, the nude mice were treated with DDP (concentration 5 mg/kg per mouse for two weeks) by injection into the tail vein. The nude mice injected A549 cells carrying an empty lentiviral vector were as the control group. The mice were euthanized and the xenograft was recovered. The tumor size and growth rate in the miR-125b-5p overexpression group were remarkably greater in contrast with the control (Fig. 5A, B). The miR-125b-5p overexpression group showed a considerable elevation in the expression of this gene (Fig. 5C), while CREB1 expression was substantially attenuated (Fig. 5D, E). The results of Ki-67 immunohistochemistry showed that tumor cell proliferation increased remarkably in the miR-125b-5p overexpression group as opposed to the control (Fig. 5F). H&E staining indicated that mitosis in tumor cells was substantially attenuated (Fig. 5F) upon overexpression of miR-125b-5p. Overall, our data indicated that miR-125b-5p was critical in the formation and development of DDP resistance in NSCLC.

### TRIM28 levels increase in A549/DDP cells, further inducing the expression of miR-125b-5p

**Fig. 6:**
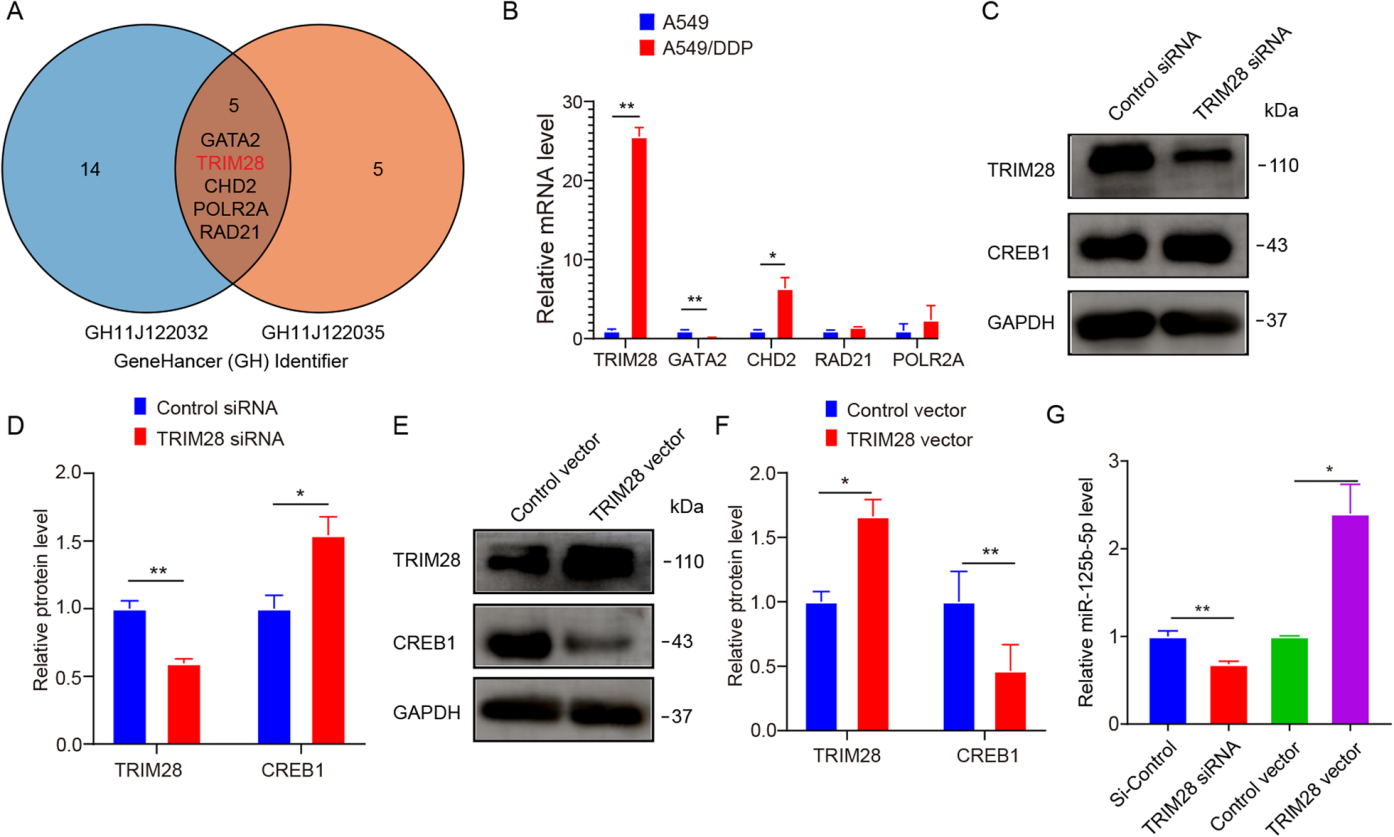
TRIM28 induces miR-125b-5p expression in A549/DDP cells. **A** Transcription factors potentially binding to the miR-125b-5p (MIR125B1) promoter were analyzed by the genecards website (https://www.genecards.org/cgi-bin/carddisp.pl?gene=MIR125B1&keywords=miR-125B), and the obtained Intersection of transcription factors in 2 Genehancer identifiers (GH11J122090 and GH11J122067) with GH score > 1.0. A total of five transcription factors potentially binding to the miR-125b-5p (MIR125B1) promoter were obtained. **B** TRIM28 had the highest expression in A549/DDP cells compare with A549 cells. **C**–**F** Western blot detects the expression level after knockdown (**C**, **D**) or overexpression of TRIM28 in A549/DDP cells (**E**, **F**). **G** The expression of miR-125b-5p was detected after knockdown or overexpression of TRIM28 in A549/DDP cells. *p < 0.05; **p < 0.01

We predicted the transcription factors regulating miR-125b-5p’s transcription using Genecard to clarify the mechanism of upstream regulation (Fig. 6A). A total of 8 predicted transcription factors were tested using qRT-PCR analysis. TRIM28 showed the highest expression in A549/DDP cells (Fig. 6B) in comparison with A549 cells. Thus, we speculated that TRIM28 may be involved in the transcriptional regulation of miR-125b-5p. Subsequently, we knocked down or overexpressed TRIM28 in A549/DDP cells, and western blotting indicated suppression (Fig. 6C, D) or elevation (Fig. 6E, F) in TRIM28 levels, respectively. And when TRIM28 was upregulated, CREB1 was downregulated in A549/DDP cells. Conversely, when TRIM28 was downregulated, CREB1 was upregulated in A549/DDP cells. After that, qRT-PCR analysis was performed to assess the expression of miR-125b-5p in each group. Overexpression of TRIM28 significantly elevated miR-125b-5p levels. However, inhibition of TRIM28 reduced miR-125b-5p levels (Fig. 6G). Thus, TRIM28 could induce miR-125b-5p expression and then affect the expression of CREB1.

### TRIM28 induces DDP resistance in NSCLC by upregulating miR-125b-5p expression

**Fig. 7:**
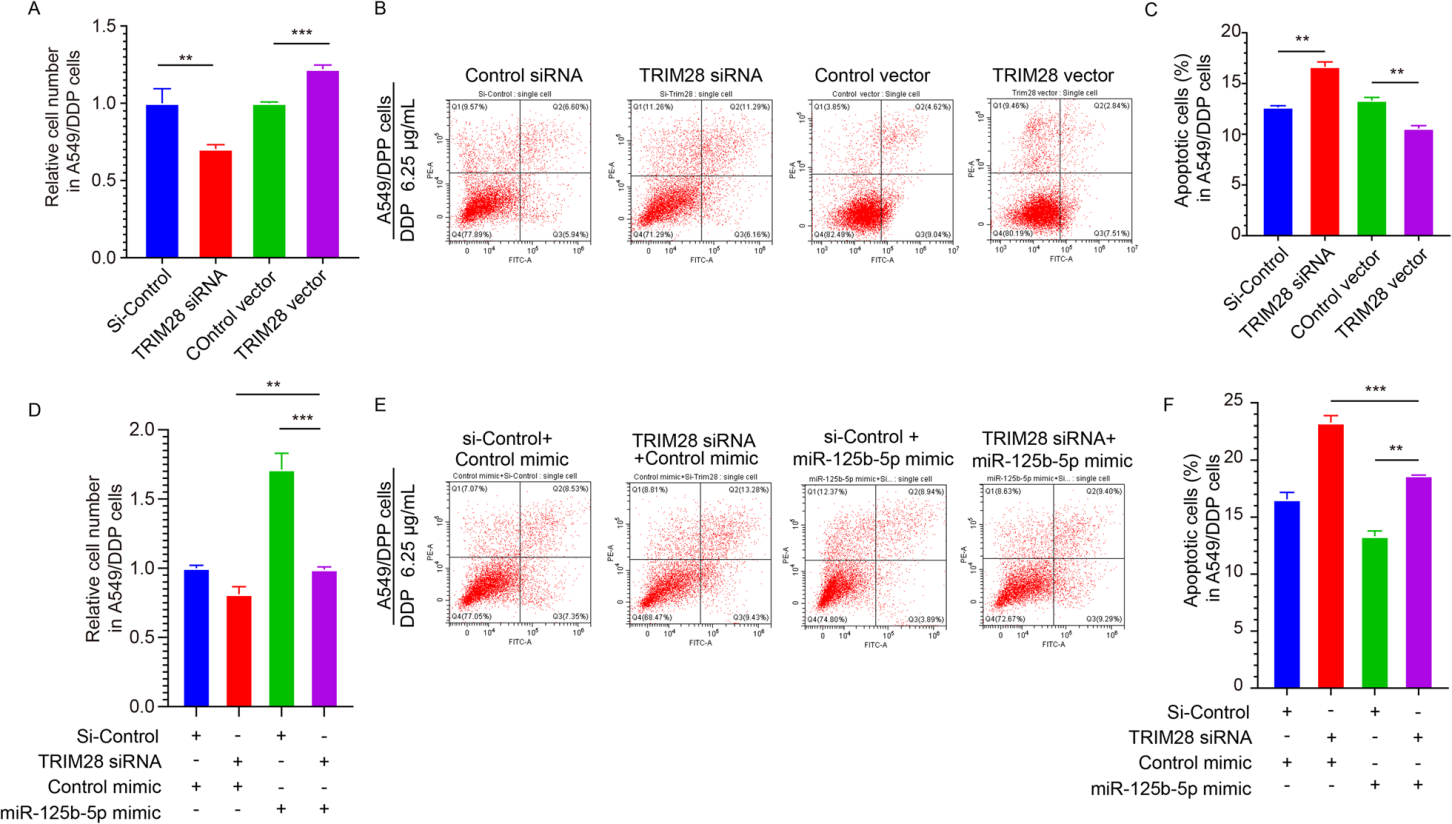
TRIM28 induces DDP resistance in NSCLC by upregulating the expression of miR-125b-5p. **A** CCK8 assay to detect DDP resistance in TRIM28 plasmid/ TRIM28 siRNA-treated A549/DDP cells. **B**, **C** Apoptosis analysis in A549/DDP cells following transfection using TRIM28 plasmid/TRIM28 siRNA upon treatment with the indicated concentrations of DDP (6.25 μg/mL). **D** CCK8 assay was used to detect DDP resistance in A549/DDP cells upon stimulation with the indicated concentrations of DDP (6.25 μg/mL) for the following conditions: a. Control mimic + Control siRNA; b. Control mimic + TRIM28 siRNA; c. miR-125b-5p mimic + Control siRNA; d. miR-125b-5p mimic + TRIM28 siRNA. **E**, **F** Apoptosis analysis for A549/DDP cells following transfection described in D with DDP. **p < 0.01, ***p < 0.001

To investigate whether TRIM28 impacted DDP resistance in NSCLC, we knocked down or overexpressed TRIM28 in A549/DDP cells. Subsequently, we analyzed the viability of these cells treated with DDP by CCK-8 assay. The findings demonstrated that the viability of the A549/DDP cells in the TRIM28 overexpression group stimulated via DDP was substantially elevated as compared to the control vector group (Fig. 7A). On the contrary, the cell viability in the TRIM28 siRNA group was considerably attenuated (Fig. 7A) relative to the control siRNA group. Additionally, we detected apoptosis in A549/DDP cells following stimulation with DDP. The rate of apoptosis in A549/DDP cells in the TRIM28 overexpression group was reduced as opposed to the control group (Fig. 7B, C), while it was increased significantly in the TRIM28 siRNA group (Fig. 7B, C). Collectively, these findings suggested that TRIM28 induced resistance to DDP in A549/DDP cells. Thus, TRIM28 influenced DDP resistance in NSCLC.

Four different groups of A549 and A549/DDP cells were transfected as follows: a. Control siRNA + Control mimic; b. TRIM28 siRNA + Control mimic; c. Control siRNA + miR-125b-5p mimic, and d. TRIM28 siRNA + miR-125b-5p mimic, to confirm whether TRIM28 induced DDP resistance in NSCLC by elevating miR-125b-5p levels (Fig. 7D–F). The cell viability in the group transfected with the TRIM28 siRNA + miR-125b-5p mimic was between that in the TRIM28 siRNA and miR-125b-5p mimic group (Fig. 7D) stimulated with DDP. Thus, the inhibition of TRIM28 expression reversed miR-125b-5p’s effects, thereby proving that, in NSCLC, TRIM28 induced DDP resistance by upregulating the expression of miR-125b-5p. Additionally, apoptosis assays also confirmed the above finding (Fig. 7E, F).

## Discussion

DDP, which initiates the apoptosis program by inducing DNA damage in tumor cells, is the common chemotherapeutic drug for the clinical treatment of NSCLC [20]. However, long-term DDP treatment can easily cause NSCLC cells to develop resistance to DDP, leading to cancer recurrence and poor prognosis in patients with NSCLC by severely limiting the chemotherapeutic efficacy [21]. The mechanism of chemical resistance to DDP is complex and involves multiple cellular processes including apoptosis [22, 23]. Thus far, DDP resistance in NSCLC remains a perplexing problem in chemotherapy. Thus, the mechanism underlying DDP resistance needs to be elucidated in NSCLC. miRNAs can induce DDP resistance in several cancer types [24–27]. The tumor suppressor effects of miR-125b-5p have been reported previously [28]. However, in NSCLC, the role and mechanism of miR-125b-5p in DDP resistance remain unknown.

In this study, we found high miR-125b-5p levels were associated with DDP resistance in NSCLC via a GEO dataset (GSE168707) analyzed. Then we found miR-125b-5p was upregulated in A549/DDP cells than that in A549 cells. And over expression of miR-125b-5p can induce DDP resistance in NSCLC cells. Whereas knocking down miR-125b-5p can inhibit DDP resistance in NSCLC cells. Then, we searched for the target gene of miR-125b-5p and verified CREB1 as a candidate. Accumulating evidence shows that CREB1 performs an integral function in cell apoptosis, proliferation, migration, and mitochondrial homeostasis [29, 30]. A recent study indicates that CREB1 mutation reduces phosphorylation levels in CREB1, leading to DDP resistance in NSCLC [17]. Herein, the results of western blotting, luciferase reporter, cell apoptosis, and cell viability assays confirmed CREB1 as a direct target of miR-125b-5p. Finally, we indicated that miR-125b-5p suppressed CREB1 expression and induced DDP resistant in xenograft mouse model. Above all, we indicated miR-125b-5p induced DDP resistance by down-regulating CREB1 expression in NSCLC.

To identify the upstream regulatory mechanism of miR-125b-5p, we screened its transcription factors. By performing qRT-PCR, TRIM28, as a transcription factor was confirmed to regulate miR-125b-5p levels. TRIM28 expression is upregulated in cancer including LC, which is also implicated in drug resistance [31]. Recent studies show that TRIM28, a chromatin-related protein, functions as a transcription factor [32]. TRIM28 can promote tumorigenesis in NSCLC cells [33]. Subsequently, via over expressed or knocked down TRIM28 in A549/DDP cells, TRIM28 can regulate miR-125b-5p expression and cause the change of CREB1 expression. Finally, we found TRIM 28 affected DDP resistance in NSCLC through regulating miR-125b-5p via cell proliferation and apoptosis assay. These studies combined with our study revealed the importance of TRIM28 as a regulator of miR-125b-5p in DDP resistance in NSCLC.

In conclusion, our results provide the first clue that in NSCLC, the TRIM28/miR-125b-5p/CREB1 axis is crucial in regulating DDP resistance. It is anticipated that the combination of targeted miR-125b-5p and cisplatin will provide an effective therapeutic option for improving the treatment efficacy of cisplatin for NSCLC. In future research, we will focus on the molecular mechanism of TRIM28 upregulation in cisplatin resistance of NSCLC.

## Supplementary Information

**Additional file 1.** CREB1 decreased the DDP resistance in A549/DDP cells.

**Additional file 2.** Three repeated original images of western blot.

## Data Availability

All the data are presented in the manuscript. If anyone needs to obtain the original data, they can contact Associate Professor Yan Liang privately by email at lyan@wnmc.edu.cn.

## Abbreviations

NSCLC: Non-small cell lung cancer
CREB1: CAMP response element binding protein 1
qRT-PCR: Quantitative reverse transcription PCR
HE staining: Hematoxylin–eosin staining
DMEM: Dulbecco’s modified eagle medium
FBS: Fetal bovine
RIPA: Radio immunoprecipitation assay lysis buffer
SDS-PAGE: Sodium dodecyl sulfate–polyacrylamide gel electrophoresis
