# Supplementary material for: miR-125b-5p upregulation by TRIM28 induces cisplatin resistance in non-small cell lung cancer through CREB1 inhibition
Source: BMC Pulm Med. 2022 Dec 7;22:469. doi: 10.1186/s12890-022-02272-9 (PMC9730690; doi:10.1186/s12890-022-02272-9)

**Additional figure 01**

**CREB1 decreased the DDP resistance in A549/DDP cells.**

**A-B.** Western blotting shows CREB1 protein expression in A549/DDP cells. Images and analyses are displayed in A-B, respectively. **C-D.** Western blotting shows protein expression after knocking down CREB1 in A549/DDP cells. Images and analyses are displayed in C-D, respectively. **E-F.** Western blotting shows protein expression after overexpressing CREB1 in A549/DDP cells. Images and analyses are displayed in E-F, respectively. **G.** CCK-8 assay to detect the effects of knocking down or overexpressing CREB1 on the viability of A549/DDP cells after cisplatin treatment. **H-I.** Apoptosis assay to detect the effects of knocking down or overexpressing CREB1 on the apoptosis rates of A549 cells following cisplatin treatment. *p <0.05, **p <0.01, ***p <0.001.


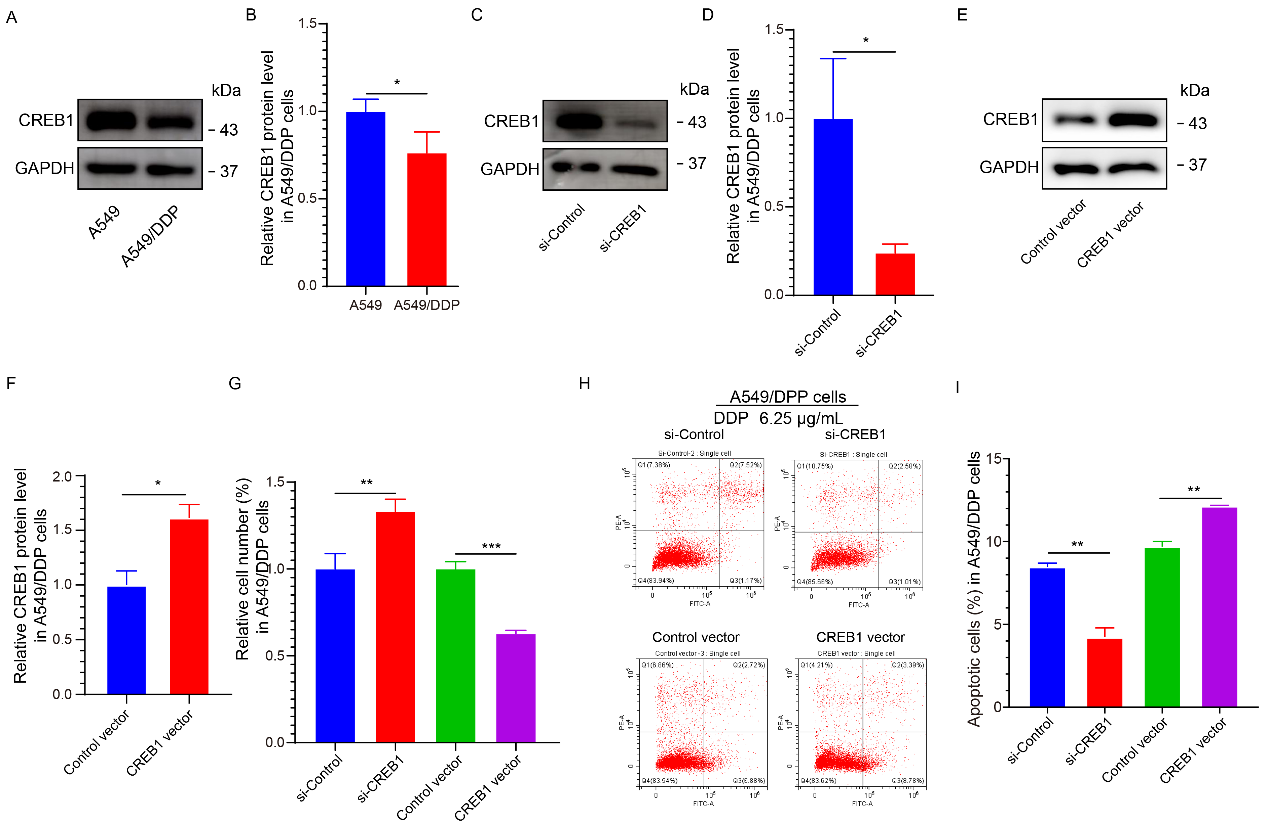

Supplement: Supplementary file 1 — Additional file 1. CREB1 decreased the DDP resistance in A549/DDP cells. [file 12890_2022_2272_MOESM1_ESM.docx]
