# Supplementary material for: miR-125b-5p upregulation by TRIM28 induces cisplatin resistance in non-small cell lung cancer through CREB1 inhibition
Source: BMC Pulm Med. 2022 Dec 7;22:469. doi: 10.1186/s12890-022-02272-9 (PMC9730690; doi:10.1186/s12890-022-02272-9)

Figure 3C. Expression of CREB1 were detected by western blot for three repeats

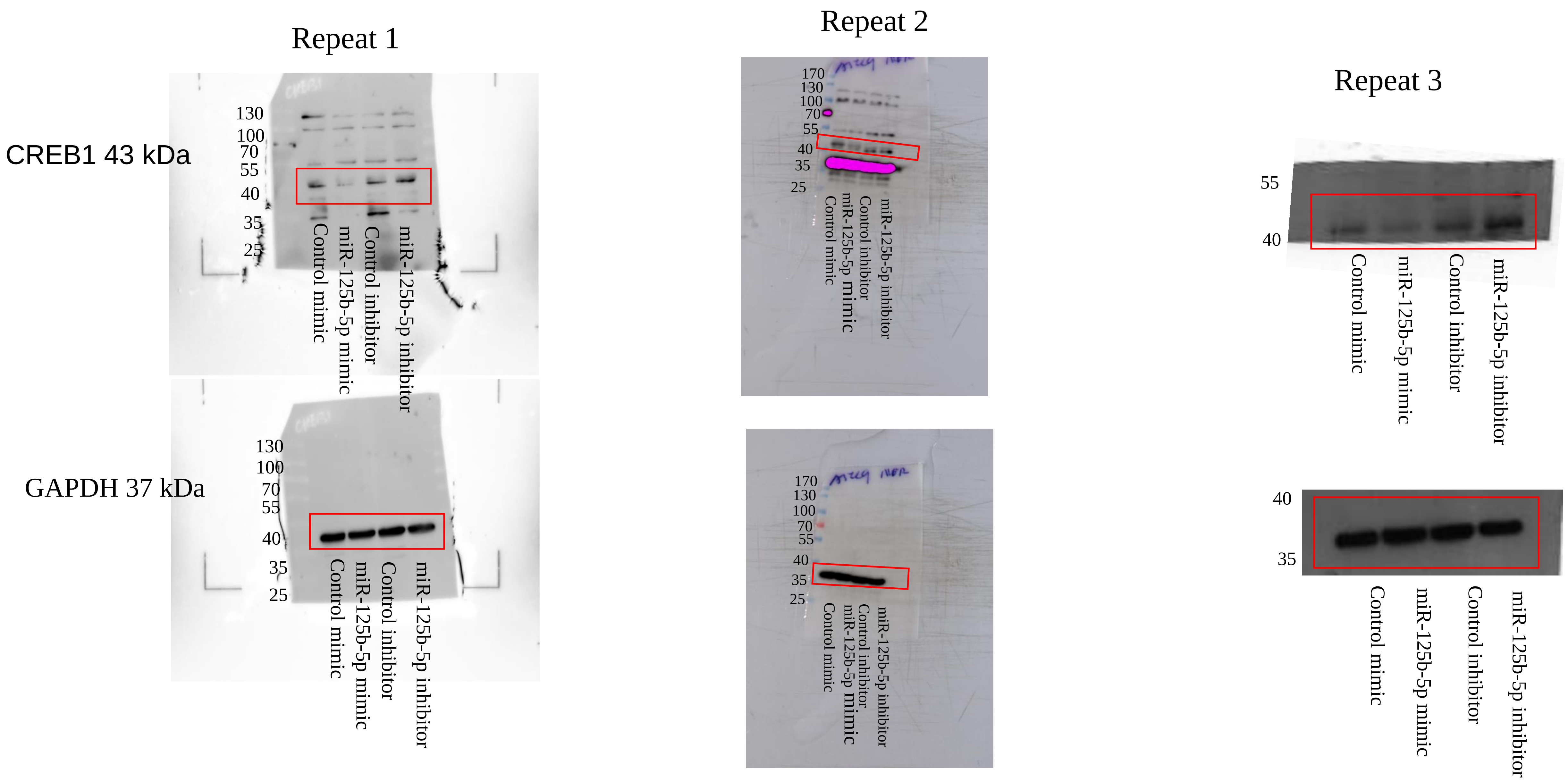

Figure 3G. Expressions of CREB1 were detected by western blot for three repeats

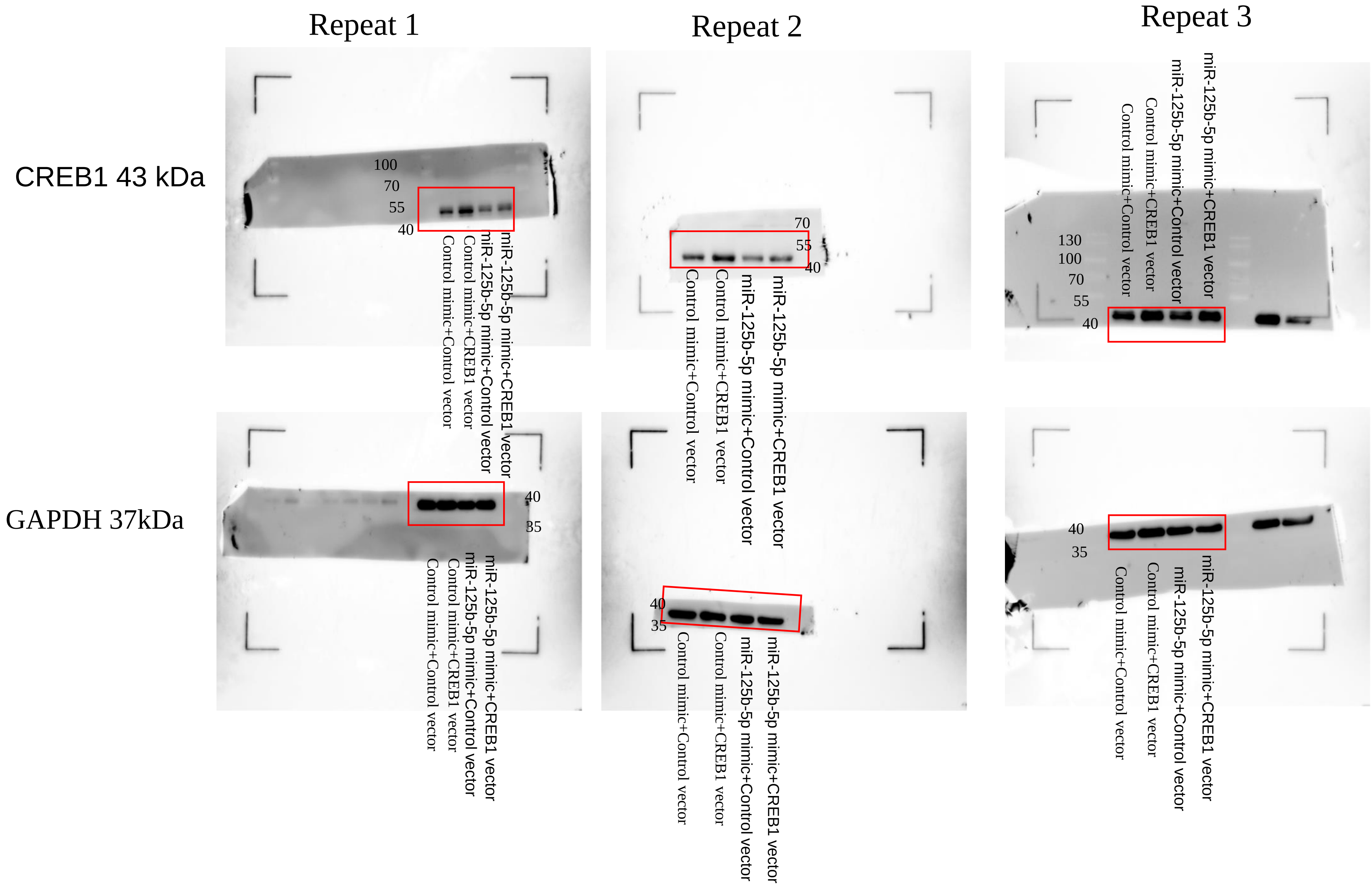

Figure 4D. Expression of CREB1 were detected by western blot for three repeats

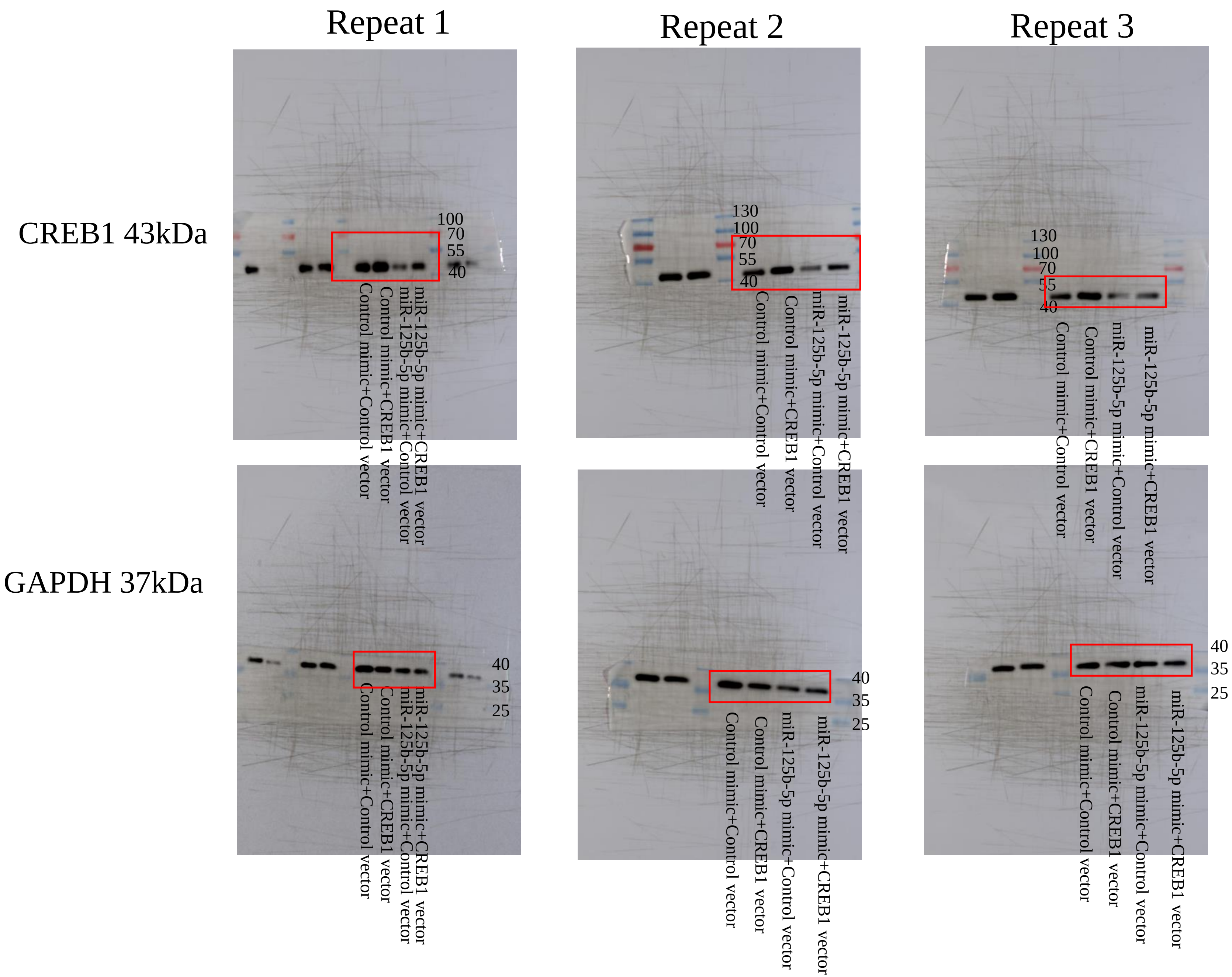

Figure 5D. Expressions of CREB1 were detected by western blot for three repeats

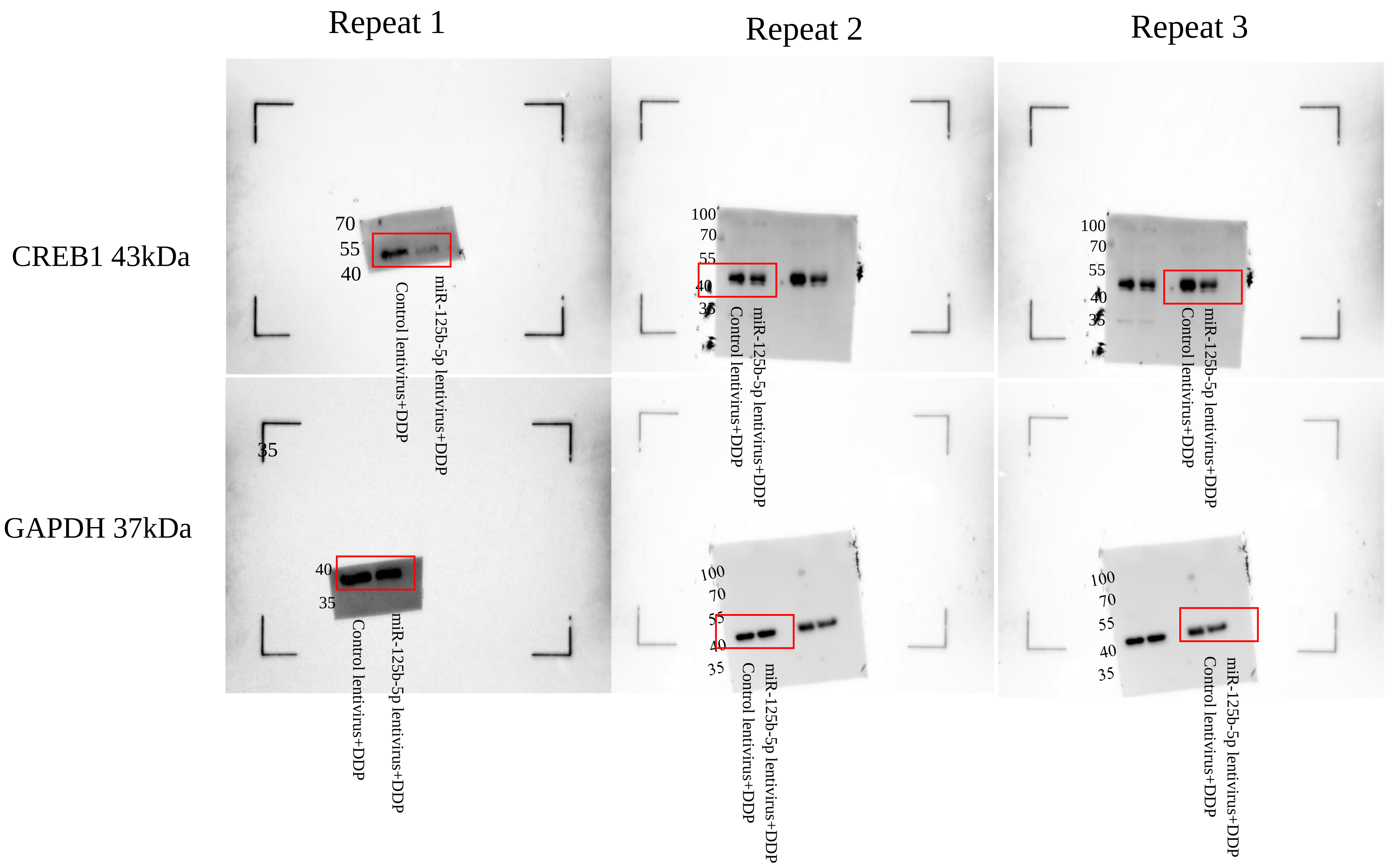

Figure 6C. Expressions of TRIM 28 and CREB1 were detected by western blot for three repeats

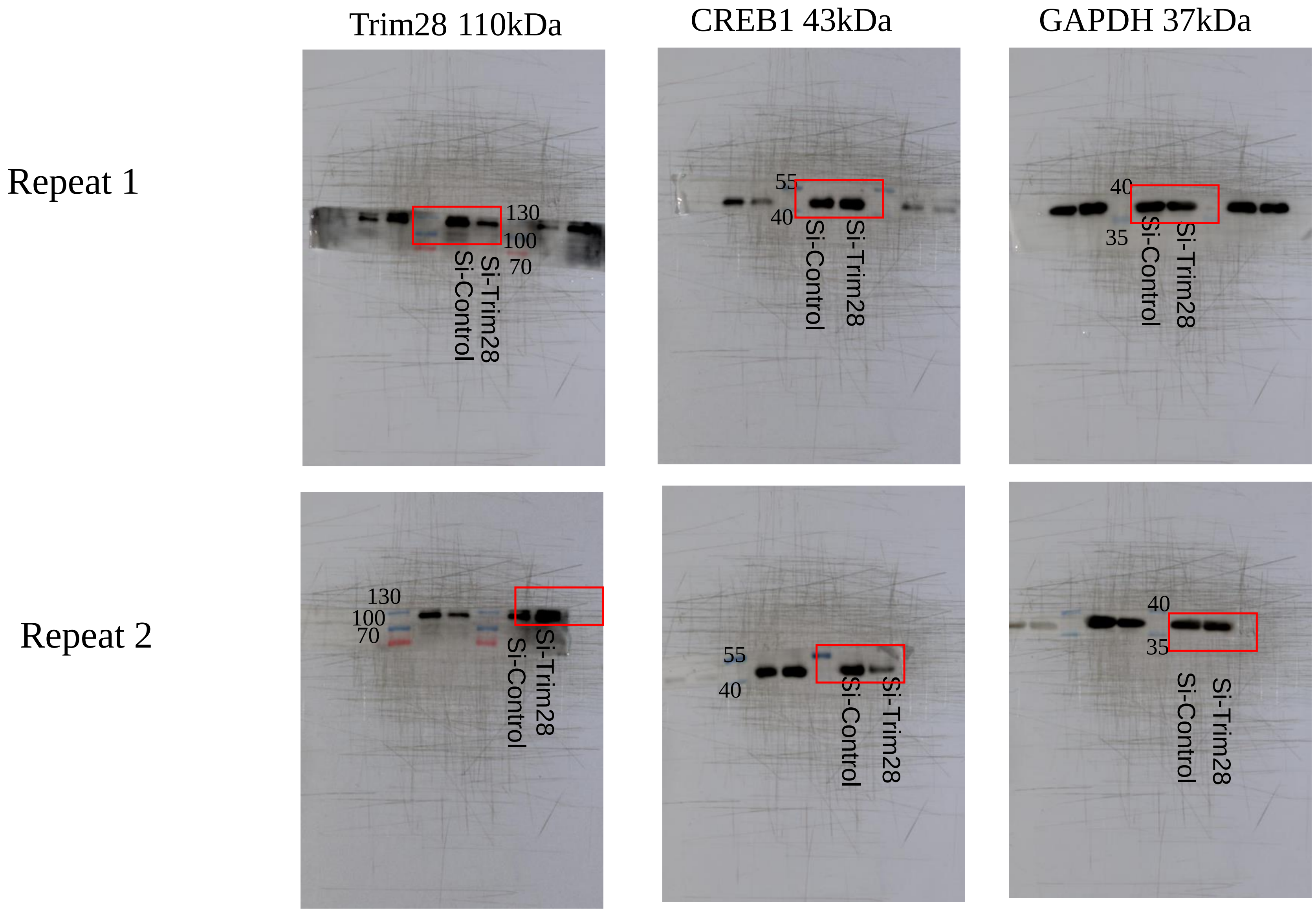

Figure 6C. Expressions of TRIM 28 and CREB1 were detected by western blot for three repeats

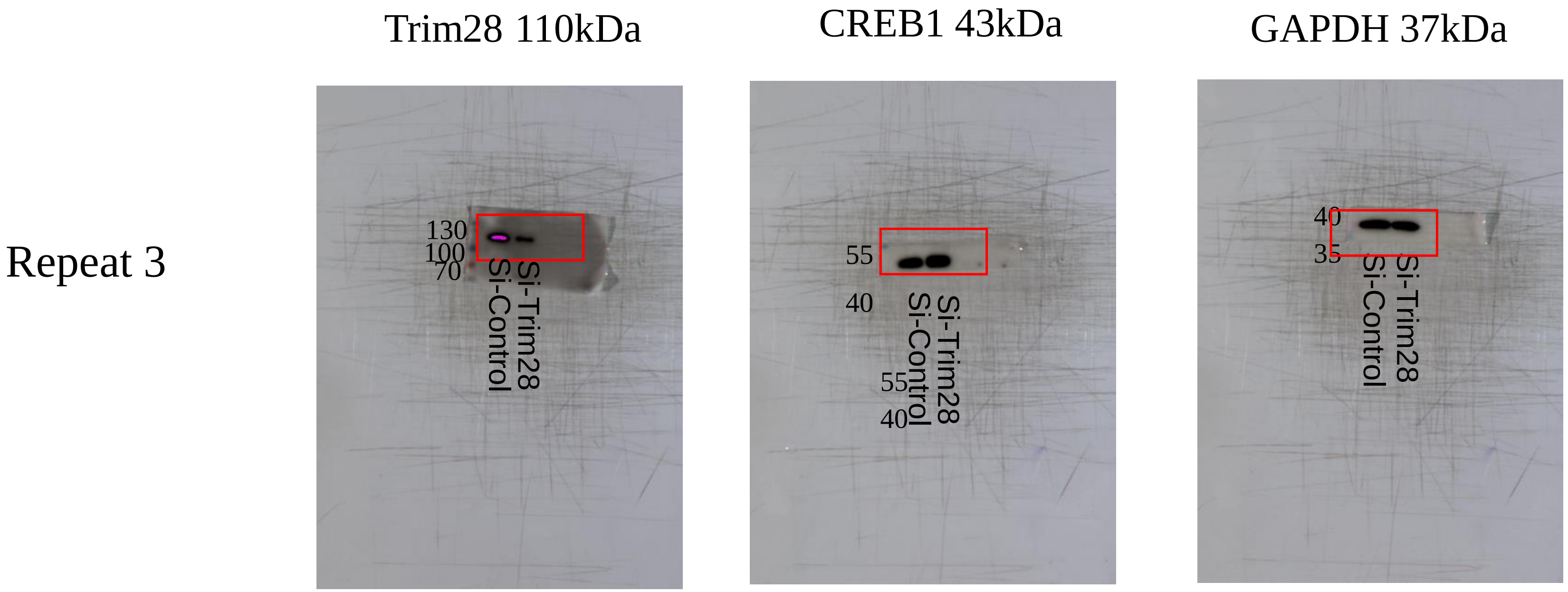

Figure 6E. Expressions of TRIM 28 and CREB1 were detected by western blot for three repeats

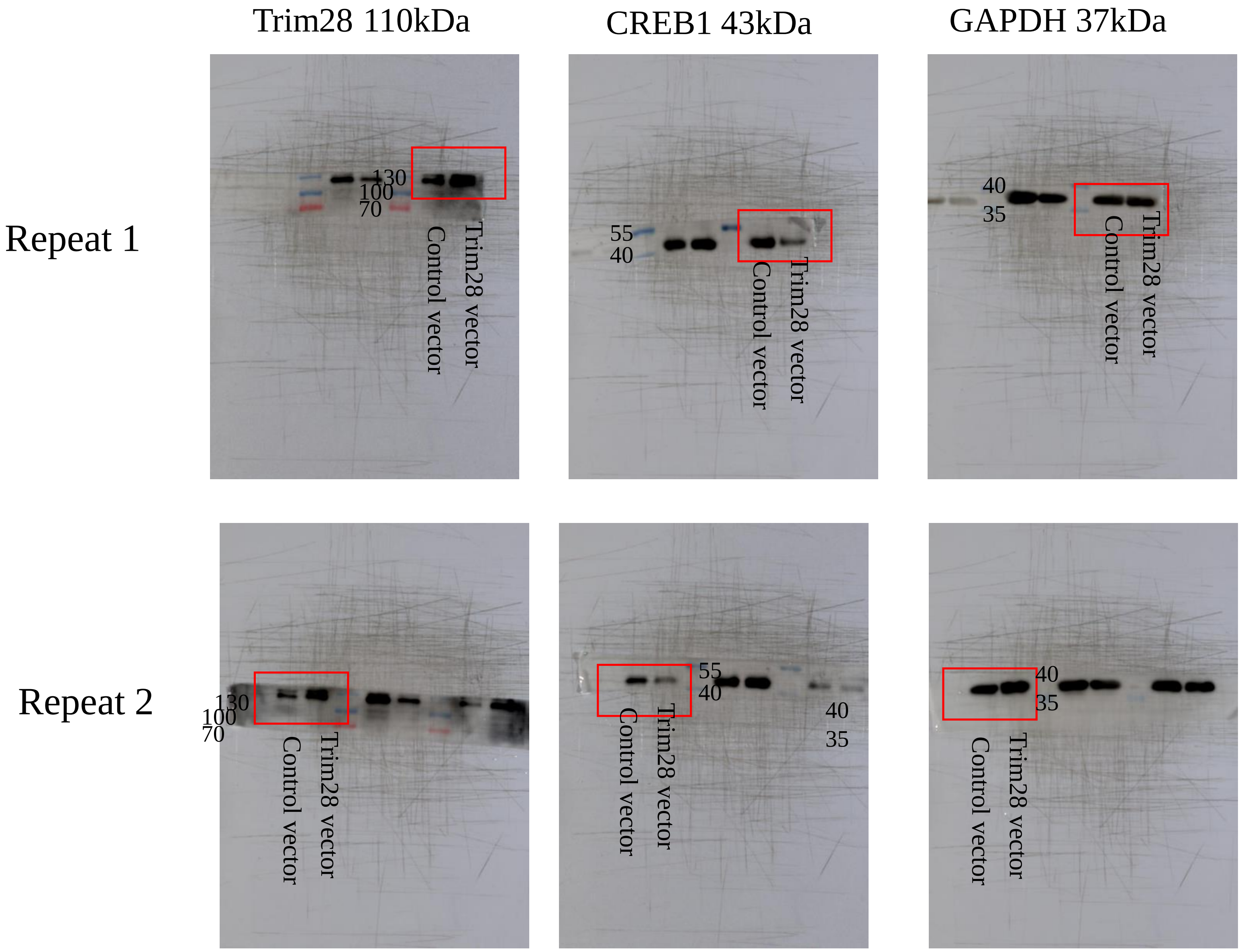

Figure 6E. Expressions of TRIM 28 and CREB1 were detected by western blot for three repeats

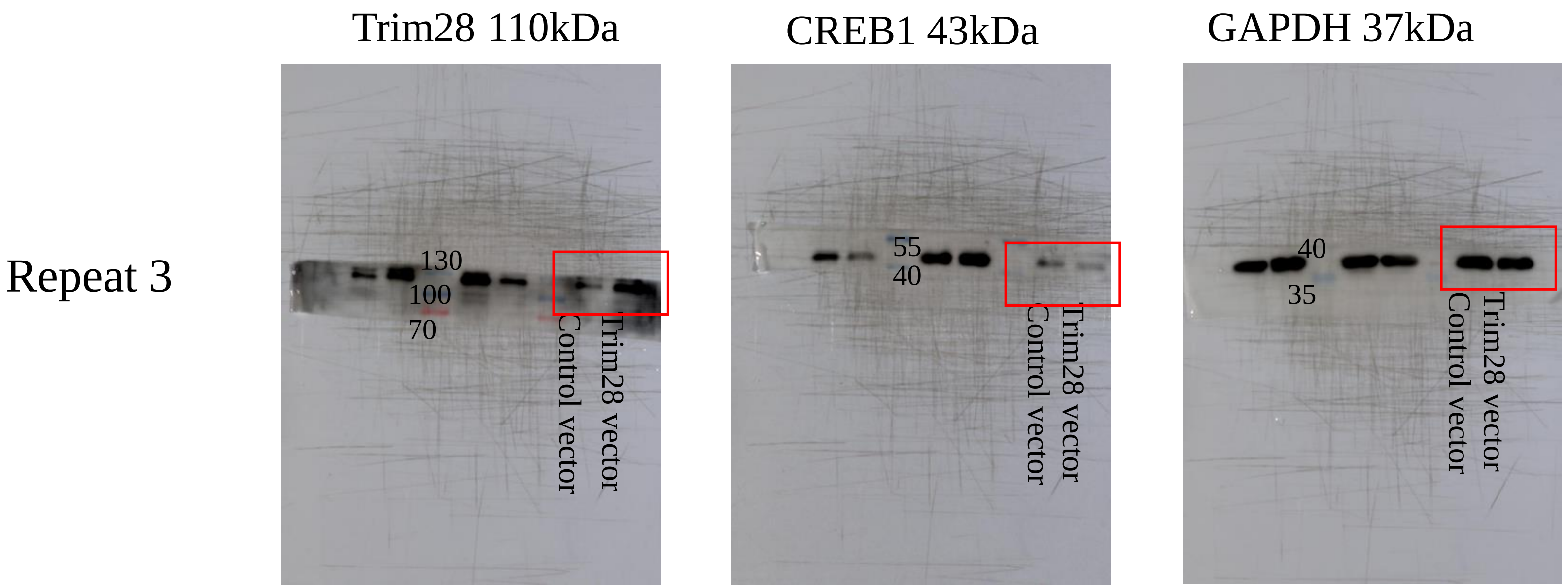

Figure S1A. Expressions of CREB1 were detected by western blot for three repeats

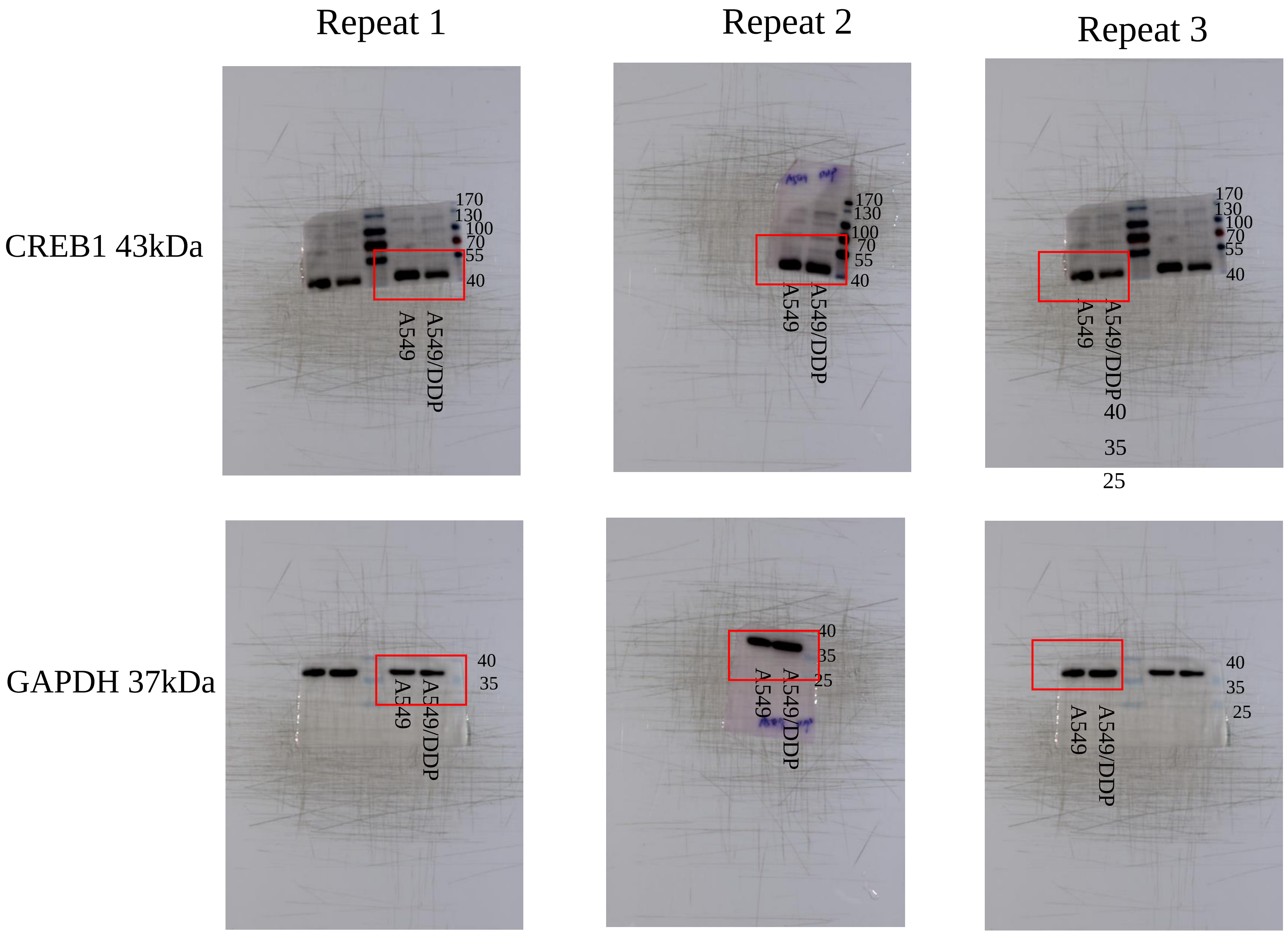

Figure S1C. Expression of CREB1 were detected by western blot for three repeats

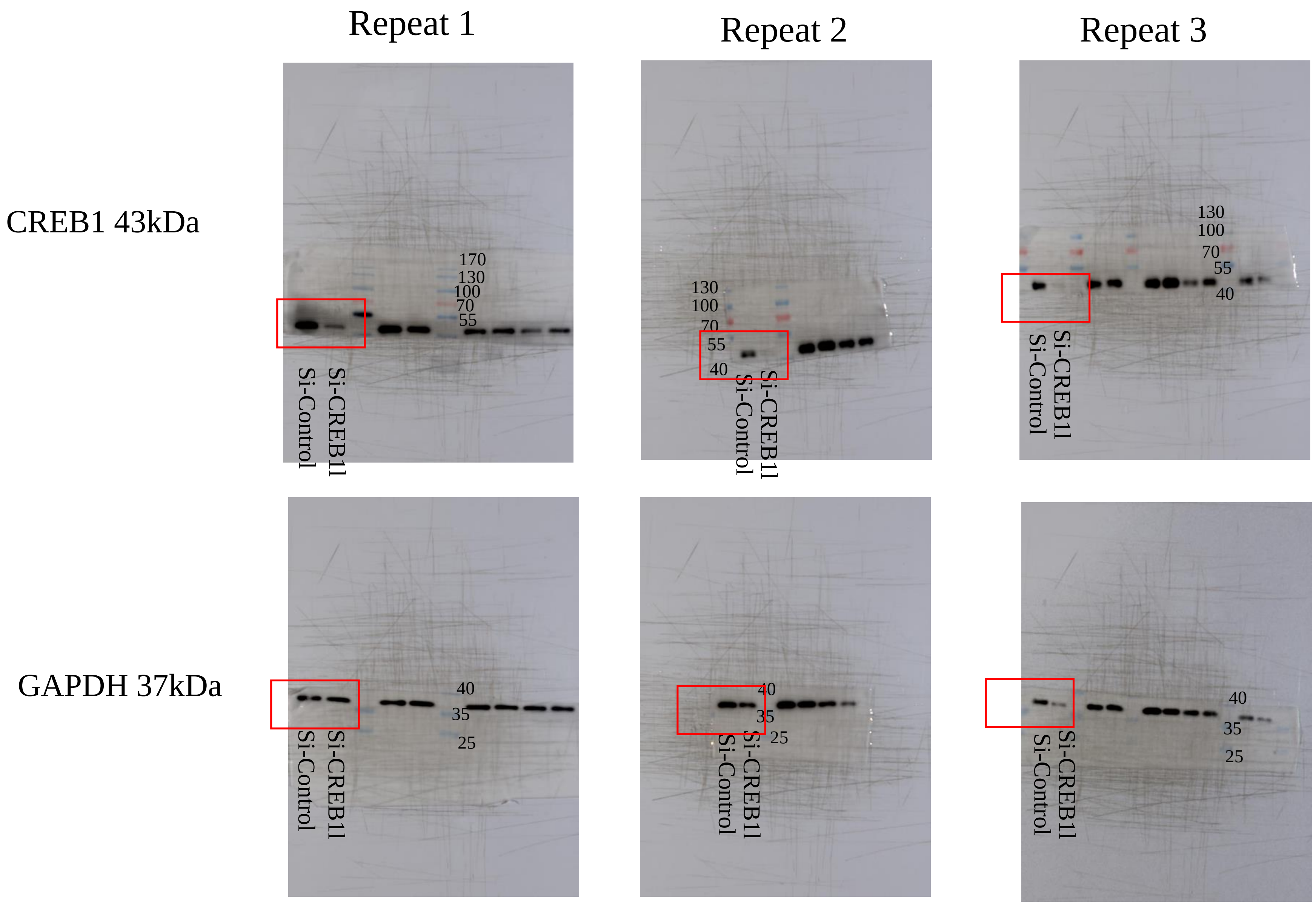

Figure S1E. Expression of CREB1 were detected by western blot for three repeats

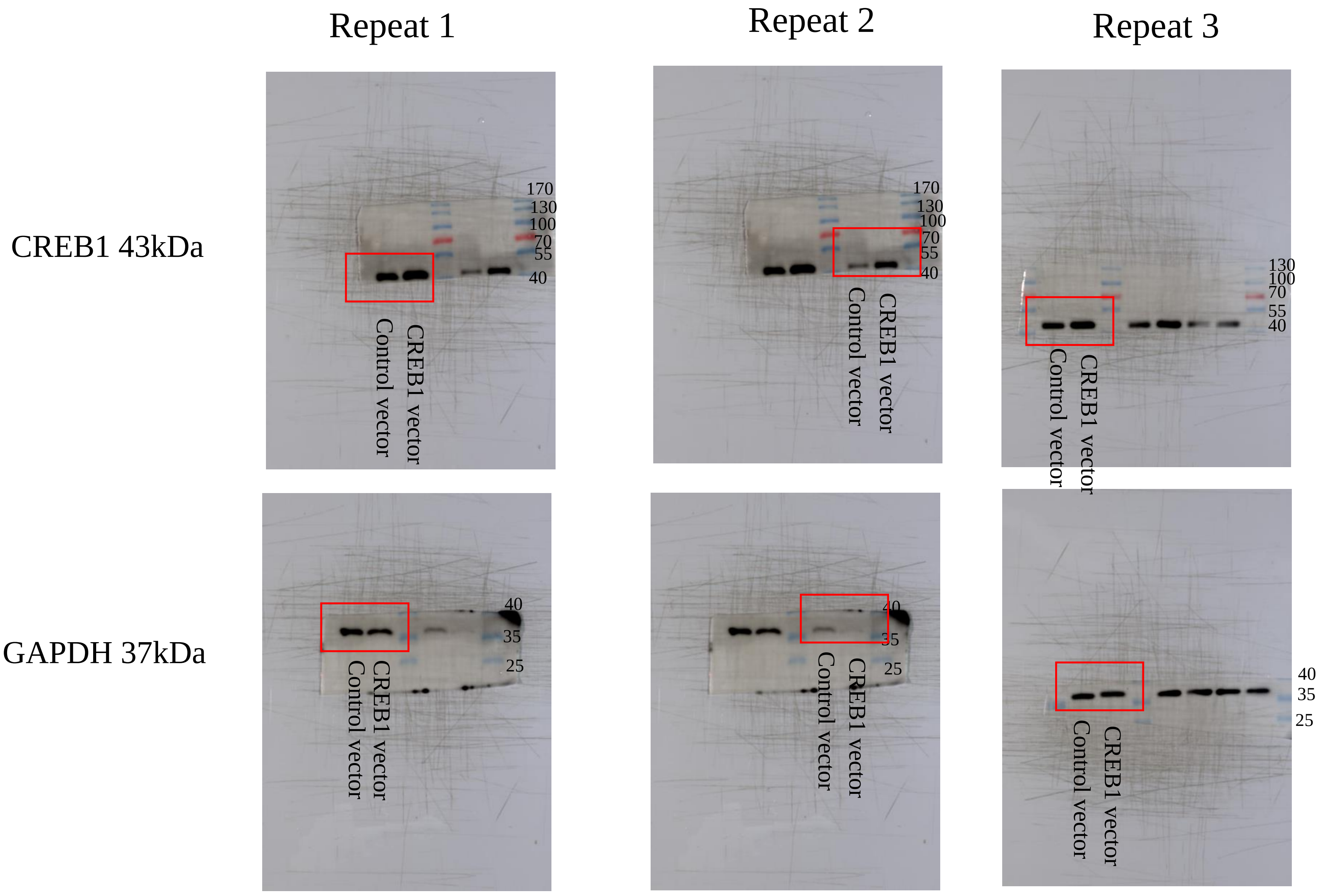

Supplement: Supplementary file 2 — Additional file 2. Three repeated original images of western blot. [file 12890_2022_2272_MOESM2_ESM.pdf]
